# The Treatment of Enteric Fever
1Read at the meeting of the Bristol Medico-Chirurgical Society, on December 8th, 1897.


**Published:** 1898-03

**Authors:** J. Michell Clarke

**Affiliations:** Physician to the Bristol General Hospital


### THE TREATMENT OF ENTERIC FEVER.[1]

BY

J. Michell Clarke, M.A., M.D. Cantab., F.R.C.P.,

*Physician to the Bristol General Hospital.*

---

The proper treatment of a fever which, as in the case of enteric fever, is due to a specific organism consists in the administration of some substance which will destroy or check the growth of the organism, and neutralise or render inert its toxic products

[1] Read at the meeting of the Bristol Medico-Chirurgical Society, on December 8th, 1897.

when they have already gained entrance to the system; but unfortunately we are not at present in possession of such a remedy, although the progress on these lines that has recently been accomplished in dealing with some other of the specific fevers, and the efforts already made in enteric fever itself, lead us to hope that we may have one in the near future. Then there will be no scope for discussion on the treatment of enteric fever, or only the very limited one afforded by the investigation of the best mode of administration of the therapeutic agent.[1]

Failing true specific treatment, our efforts must then at present be concerned, since we cannot meet and destroy the virus by its proper antidote, with the best means of sustaining the patient's strength, of increasing and maintaining his powers of resistance against the toxic products absorbed from the intestine; of reducing fever when this is excessive in itself; of hindering the fermentative processes going on in the intestine, and thus preventing as far as possible the absorption of poisonous products which can so readily take place through the broken surface of the ulcerated intestinal wall; and of combating those complications which so frequently arise in the later stages of the disease and are in themselves directly dangerous to life. I propose to deal chiefly with the best means of maintaining the general well-being, of treating fever, of preventing excessive fermentation processes in the intestines, and of dealing with the most serious complications.

With regard to the general management of the patient and the hygienic measures to be adopted I need say little. Absolute rest with a fluid, easily assimilable, and nourishing diet are of course essential. Milk, which may require to be modified in different ways to suit individual patients, will be generally accepted as the best food, with the addition of barley-water, beef-tea, and meat-extracts; I allow in addition the beaten up

[1] With regard to the prophylaxis of enteric fever attempts have been made by Wright and Semple and Pfeiffer and Kolle to "vaccinate" against enteric fever by injections of emulsions of Agar cultures of typhoid bacilli, in which the bacilli have been killed by heating to 60° C for five minutes, based on the view that Widal's serum-reaction is a sign of immunity. The process is free from risk, but is still on its trial.

yolk of an egg, once or twice in the twenty-four hours, but I think that the use of more solid food, which has been recently advocated in order to obviate the extreme weakness of the late periods of the illness and to shorten the period of convalescence, is not at all advisable. It must largely increase the risk of perforation and hemorrhage, those two most formidable complications.

As to the use of alcohol, the guide to its administration is to be found in the state of the pulse. Alcohol should be given freely when there is any sign of heart-failure, reducing or dropping the dose again when the threatening symptoms are averted. It is indicated in states of quiet delirium, when collapse is threatened, and in insomnia; a sufficient dose of alcohol at bed-time often ensures a good night. It is also of service in the apyrexial interval before solid food can be allowed, as it mitigates to some extent the pangs of hunger, besides being a useful stimulant at a time when the action of the heart is often weak.

Perhaps the chief differences of opinion lie in the estimation of the relative advantages of the expectant, antipyretic, and antiseptic methods of treatment, or of the combination of the two latter. Taking first of all antipyretic treatment, it is to be borne in mind that in treating fever we deal only with a symptom and not with the disease, and that a certain degree of pyrexia is considered by many to be not only not injurious, but beneficial to the organism in hindering the growth and development of the virus.[1] But although opinions may differ as to the

[1] The *Practitioner*, 1897, lix. 625, quotes some interesting experiments on this subject which have recently been made. Loewy and Richter (*Arch. f. path. Anat.* [etc.], 1896, cxlv. 49) raised the temperature of rabbits, by puncture of the heat-regulating centre in the corpus striatum, sometimes as high as 107.5° F. The temperature remained high for months, or if it fell, could be raised again by a fresh puncture, and the rabbits were well in other respects. They were inoculated with pneumococci and with the organisms of fowl-cholera, swine-erysipelas and diphtheria, and the course of these infections was always favourably influenced. None of the animals died sooner than the control animals, and some survived what should have been a fatal dose. Fischl (*Prag. med. Wchnschr.*, 1897, xxii. 49, 63) on the other hand found that when the temperature of rabbits was artificially lowered to 10° C. they became more susceptible to injections of cultures of pneumococci and died more readily than the control animals.

utility of reducing pyrexia as an end in itself, it is generally held that a long-continued high temperature is injurious to the organism, and this view is based on the *post-mortem* changes found in the organs as a consequence of persistent high fever, which therefore calls for active interference. What mode should this interference take? "The researches of Zuntz show conclusively that the only reflex apparatus for regulating temperature *which is always at work* is that which governs the loss of heat at the surface."[1] This would indicate that the best and safest method of reducing pyrexia is that of abstracting heat from the surface by the application of cold, and clinically this is conclusively shown by the good effect of cold baths in hyperpyrexia.

The use of antipyretic drugs to reduce fever, phenacetin, kairin, antifebrin, antipyrin, *et hoc genus omne*, from which a few years ago so much was anticipated, but which has now been practically given up, is fraught with danger, because of their depressing action on the nervous system and the circulation, and because of the reduction of the flow of urine and consequent elimination of toxic products; whilst their effect on the temperature is often to lower it too suddenly, and also to be too transient. The mortality of cases in which treatment by these antipyretic drugs is carried out is from 12 to 16 per cent., that of cases treated by cold baths 6 to 8 per cent.; a result which speaks for itself.

If then it is allowed that application of cold to the surface is the best measure to take for reduction of fever, how can it be most effectually carried out? There is no doubt that by far the most effectual method is the cold bath treatment. Now in employing cold baths we are not open to the reproach of treating merely the symptom, pyrexia; for Roque and Weil claim to have shown that in "typhoid fever left to itself the toxic products manufactured by the bacillus and organism are eliminated in part during the illness. The urotoxic coefficient is double the normal, but this elimination is incomplete and it is only completed during convalescence, for the hypertoxicity continues

[1] Burdon-Sanderson in *A System of Medicine*, edited by T. Clifford Allbutt, 1896, vol. i., p. 159.

for four or five weeks after the cessation of the fever. In typhoid fever treated by cold baths the elimination of toxic products is enormous during the illness. The urotoxic coefficient is five or six times the normal. The hypertoxicity diminishes as the general symptoms mend and as the temperature falls, so that when the period of pyrexia [ends] and convalescence sets in the elimination of toxins has ceased."[1]

Further, apart from the question of treating pyrexia, the advantages claimed for the bath, and, so far as my experience goes, justly claimed, are[2] that (1) the intellect becomes clearer, the stupor lessens and the muscular twitchings disappear; (2) a general tonic action on the nervous system and particularly on the heart; (3) the insomnia is lessened, the patient usually falling asleep after each bath; and last and most important, the mortality is reduced. Thus Dr. Dreschfeld quotes:[3]

| | | | | |
|---|---|---|---|---|
| Drasche (Vienna) as finding a reduction | from | 16.2 % to 9.3 % | mortality. | |
| Tripier and Bouveret (Lyon) | ,, | 25.0 % ,, 7.5 % | ,, | |
| Osler (Baltimore) | ,, | 21.8 % ,, 7.4 % | ,, | |
| Thompson (New York) | ,, | 19.0 % ,, 7.0 % | ,, | |
| Vogl (Munich Garrison) from | 1841–1860 | mortality | 21.0 % | expectant treatment. |
| | 1860–1875 | ,, | 15.2 % | *partial* bath ,, |
| | 1875–1882 | ,, | 6.5 % | bath treatment. |

Dr. F. E. Hare[4] gives the following striking statistics of the cold bath treatment of enteric fever at the Brisbane Hospital: The mortality was lowered from 14.5 per cent. to 7.5 per cent. The number of patients was 1,902. Mortality under bath treatment—

| | | | | |
|---|---|---|---|---|
| From perforation, 2.9 %. | Before bath treatment, | | 2.9 %. |
| ,, hemorrhage, 1.2 %. | ,, | ,, | 1.88 %. |
| ,, other causes, 3.4 %. | ,, | ,, | 9.73 %. |

The death-rate per number of patients affected with enteric fever is still for England the same that Murchison gives, 17 per cent.;[5] at a large London hospital it was 26 per cent., 12 per cent., and 11.2 per cent. for the last three years respectively.

[1] *The Means by which the Temperature of the Body is Maintained in Health and Disease*, by W. Hale White, 1897, p. 73.
[2] *The Principles and Practice of Medicine*, by W. Osler, 1892, p. 35.
[3] *A System of Medicine*, edited by T. Clifford Allbutt, 1896, vol. i., p. 845.
[4] *Practitioner*, 1897, lix. 254. [5] Dreschfeld, *loc. cit.*

Dr. Dreschfeld concludes that the evidence appears to be conclusive that the hydropathic treatment strictly carried out has considerably lessened the mortality; and if I bring forward facts already familiar, my excuse must be that in view of this lowered mortality the bath treatment has not sufficiently come into use in England. I do not think the bath necessary for very mild cases in which the highest range of temperature is from 102° to 103°: for these, sponging is sufficient. I began to employ the bath treatment systematically about two and a half years ago in consequence of the favourable reports published of it, and of the fact that it is difficult to reduce the temperature sufficiently by sponging, and that the effect produced does not as a rule last long. Before this I employed cold sponging in all my cases, sponging being carried out every four to five hours as a matter of routine, except where the case was a very mild one, when it was done less often. In all others than mild cases the bath is employed; the advantages over sponging are: (1) it has greater and more prolonged effect; (2) the temperature can always be reduced by it, whereas sponging may fail; (3) the beneficial effect on the nervous and vascular systems is much more marked; (4) if properly carried out it does not necessarily involve more movement of the patient.

This last statement, which is obviously one of practical importance, may seem unlikely; and to ensure the bath being properly given with the least possible movement of the patient, if he is an adult of about the average weight, three persons will be required to lift him into the bath: if the patient is very light, two nurses can easily lift him in and out. Several times I found that the temperature did not fall in the bath, and once or twice even went on rising; but, on taking the temperature half an hour afterwards, it had invariably fallen. On only one occasion the bath failed to reduce a high temperature of 105°, and then the time of fifteen minutes was not exceeded on account of the patient's pulse being very weak. On one occasion only some collapse was produced; but then the patient was kept in the bath too long. The effect on the pulse when this is very rapid and feeble is, as a rule, good, the pulse becoming fuller and slower afterwards. I need not enter into the details of carrying out the bath treatment, as they are now well known. The bath seems especially valuable in those cases in the third and fourth week in which the temperature shows a decided tendency to rise and to remain at a high level; the effect, in cases with pronounced nervous phenomena, and with insomnia, and as said above, on the circulatory system is, as a rule, very satisfactory. In any case of enteric fever with a high temperature range, I should feel that without the bath the best was not being done for the patient. If it could not be carried out, I think sponging in ice-cold water or an ice pack is the most efficient substitute. Another measure, but less efficient, is the suspending of small pails of ice from a cradle with the patient lightly covered. The most important contra-indications of the bath are hemorrhage in the later stages, peritonitis, perforation, and possibly myocarditis. Neither pneumonia nor nephritis precludes its use. An interesting case of enteric fever with the latter complication was recently published,[1] in which Dr. Tyson showed that the amount of urine and quantity of urea excreted increased under the use of cold baths.

On the other hand, it is useless to deny that there are considerable difficulties in carrying out the bath treatment, especially when the patient is a large, heavy person. In institutions it is more easily carried out than in private, although in the houses of the well-to-do where one has plenty of assistance it can be managed without much difficulty. One objection is the great dislike that many patients have of it, the dread that it may excite in a very nervous patient may have a prejudicial effect on the course of the illness and prevent its employment. I have had patients, however, who liked it, because they felt refreshed by it. I have heard of a few cases in which the use of the bath was discontinued on account of its being followed by depression of the circulation and excessive cyanosis: but I have not met with any instance myself in which, when carefully carried out, the effect on the heart and circulation was otherwise than beneficial; and the absence of dicrotism in the pulse is noticeable in cases treated

[1] *Am. J. M. Sc.*, 1897, cxiv. 290.

on this plan. The improvement in patients who are lying in a state of stupor or semi-coma is also remarkable, and not attained by other modes of treatment. Lastly, it is of great advantage to have a nurse who has had experience in carrying out the bath treatment: this is, in my opinion, a very important point.

I believe that a further means of treatment of value lies in the administration of intestinal antiseptics. Although we cannot expect by the use of intestinal antiseptics to destroy the typhoid bacilli which, by the time treatment has begun, have already entered the intestinal wall, and reached the glands and other internal organs, we may hope to hinder the action of other organisms in the intestinal contents, whose growth is favoured during the course of enteric fever, and thereby to diminish fermentative action, undue distension of intestines from products of fermentation, and to neutralise or hinder the absorption of the poisonous products through the ulcerated surface.

Under this head I include the administration of calomel, a measure of undoubted value in the early stages. If a case is seen before the seventh day it is well to begin treatment for two or three nights in succession with a dose of gr. iij.—gr. v. of calomel. It has been claimed that the course of the fever is thus rendered milder and the duration shorter. Such a dose will usually have a mild purgative action, and thus get rid of a certain amount of toxic material, and is in addition a powerful antiseptic, for it has been shown experimentally that calomel kills bacteria, hinders the butyric acid fermentation and the formation of indol and skatol, the products of decomposition in the intestines. But for continued use some other substance must be sought, which, to be an efficient antiseptic, and at the same time innocuous to the patient, should have the following properties: It should hinder the development of bacteria and neutralise their products, and at the same time not interfere with the digestion of food: it should be insoluble, so that it will not be absorbed, and thus pass more or less unchanged through the alimentary canal, so as to exert its antiseptic action throughout the greater part of it. There is no substance known at present which fulfils all these indications; but of those that we have I believe the naphthols most nearly do so. They are nearly insoluble in water, and have at any rate no injurious effect on the organism. In a former paper[1] I showed that although naphthol $\beta$ in a proportion as large as would be present in the stomach during its medicinal administration delayed the digestion of meat, it had no effect on the digestion of milk by artificial gastric juice, and would therefore not be prejudicial in this respect in the treatment of typhoid fever.

Drs. N. Surveyor and Vaughan Harley state[2] with regard to the antiseptic power of $\beta$-naphthol, from test-tube experiments, that both $\beta$-naphthol and bismuth subnitrate have the power of preventing or hindering the growth of various micro-organisms, and $\beta$-naphthol is the stronger of the two in some cases; the safest proportion for $\beta$-naphthol being 8—1000, and for bismuth subnitrate 10—1000. Further experiments on the number of colonies present in different parts of the intestines of dogs fed on the same diet showed that bismuth subnitrate is more powerful as a germicide than $\beta$-naphthol when large doses are used, but internally $\beta$-naphthol appears to act better than bismuth subnitrate. The reason for this discrepancy they find in the fact that although the latter is the stronger antiseptic, its value is lowered on account of its constipating property.

Mr. F. W. Stoddart kindly made some plate cultivations for me from the stools of patients with enteric fever who were being treated by hydronaphthol and bismuth $\beta$-naphthol. Although from the complicated nature of the conditions present it is impossible to draw any conclusions from the number of colonies present, which were enormous, there was one marked difference from stools of enteric fever patients not treated by antiseptics. This difference lay in the absence of colonies of liquefying organisms, which did not appear for fifteen days in one case. Mr. Stoddart tells me that these liquefying organisms are the most sensitive to the action of antiseptic agents, and so far as it goes this affords evidence that the naphthols have some antiseptic action in the intestinal contents. One case was

[1] *Practitioner*, 1890, xlv. 1.

[2] "The Action of Beta-Naphthol and Bismuth Subnitrate as Intestinal Antiseptics," *Brit. M. J.*, 1895, ii. 1483.

treated by baths, the other not; there was no obvious difference between the character of the cultivations.

If any continuous efficient intestinal antisepsis is to be produced, the dose must be frequently given in order to keep up a constant effect: I usually give gr. iii.—v. of bismuth $\beta$-naphthol or hydronaphthol every 2½ to 3 hours, day and night. Unfortunately the naphthols leave a pungent after-taste; this is least with bismuth $\beta$-naphthol: the dose is given floated out on a little milk. Clinical evidence seems to show that bismuth $\beta$-naphthol is the most useful, it has very little taste and is the most effective in checking diarrhœa. Under its action the stools lose their offensive odour and become dark in colour. The diarrhœa of enteric fever is soon checked by its administration, but if it should continue a very efficient combination is formed by the addition to each dose of gr. i.—gr. ii. of Dover's powder. Besides the marked effect on diarrhœa and odour of the stools, hydronaphthol and bismuth $\beta$-naphthol have a most important action in preventing excessive abdominal distension; in fact, this symptom, which under former methods of treatment was often so troublesome, is as a general rule absent when these antiseptics are given. This seems to me an important result and a considerable element in the successful management of enteric fever, for excessive distension is dangerous in many ways. First, by favouring the occurrence of the most formidable and fatal complication, perforation of the intestine, which according to Murchison occurs in nearly one-fifth of the fatal cases, and 1 in 33 of all cases. Then when the heart's action is weak it must be further embarrassed and the descent of the diaphragm impeded by the pressure from over-distended intestines.

In my former paper,[1] besides the above advantages, I further claimed for the use of the naphthols that the tongue remains cleaner, relapses are less frequent, the duration of the disease is shorter, and infection to others less probable. More extended experience has, however, shown that with the exception of the tongue being cleaner, no advantage in the other respects is obtained over other methods of treatment.

[1] *Loc. cit.*

Other antiseptics that have been advocated are carbolic acid, liq. hydrarg. perchlor., salol, betol, benzonaphthol, and chlorine. It is difficult to use the first two in sufficient doses without running the risk of producing toxic effects. Salol is efficient, but has the disadvantage of giving rise in the intestine to salicylic acid, which passes out by the kidneys, and is probably irritating, and may be injurious to them when excreted in large doses for some time. Benzonaphthol seems to me less useful than the others.

It is difficult to form an estimate of the effect of the antiseptic treatment on the mortality. Dr. Dreschfeld states[1] that at the Monsall Fever Hospital, where the mean mortality is about 17 per cent., the mortality fell during one year when this treatment was extensively used to 13 per cent.

With regard to the treatment of special symptoms or complications a few words may be said. Small doses of phenacetin and caffeine were found to be very efficacious in relieving the headache of the early stage when this was severe. When there is much delirium the application of cold water to the head by means of Leiter's tubes is often effectual: an increase in the amount of stimulant taken is generally necessary, and a dose of morphia or chloral and bromide may be required.

The treatment of abdominal distension and of diarrhœa has been already referred to: should the latter not be controlled by the measures there recommended, a starch and opium enema generally stops it. For constipation I have always found small enemata of soap and water, or of soap and water mixed with olive oil, sufficient. Insomnia is generally relieved by bathing or an extra dose of stimulant at night; if more is required, a dose of morphia is in my experience the best remedy. Close attention to the state of the heart and pulse is most important in the later stages of the fever: should any threatening of heart-failure appear, the patient should at once be put upon digitalis; and if evidence of heart-failure is actually present, hypodermic injections of strychnine are the most effectual remedy. Caffeine is also extremely valuable in this condition, it may be given by the mouth, or in case of pressing need injected subcutaneously.

[1] *Loc. cit.*, p. 846.

Caffeine and strychnine may both be continued for some time till the danger is past with beneficial effects. I have also found hypodermic injections of spartein sulphate (gr. ½) of great service when one wished to produce a rapid effect upon the heart. Of course when digitalis is given for any time its effects need to be carefully watched. For hemorrhage, besides the ordinary measures in the way of regulation of food, application of ice to the abdomen, ice to suck, and administration of morphia, turpentine in capsule in doses of 10—20 minims every 4 hours has given me good results, or lead and opium may be administered. If the post-hemorrhagic anæmia is extreme, subcutaneous or intravenous injections of saline fluid may be required. One boy, in whom after a large hemorrhage in the first week the extremely anæmic condition gave rise to anxiety, seemed to derive benefit from marrol, a preparation of the red marrow of the ox.

I may perhaps give a brief account of the cases under my care, though the number is not a large one, since March, 1893, which were treated on the above plan.

The number of cases is 70, of these about 30 were treated before I began to use the bath. In these cold sponging was carried out every 4 to 6 hours, except in the mild cases, when it was done less often. Of the others, sponging was resorted to in very mild, the bath treatment in the moderate and severe, cases. The cases were of all grades of severity, as is roughly shown by the temperature records: in 41 cases the temperature at some time exceeded 104°, in 15 it reached 105°, in 4 106°, and in 1 107°: of the last 5, 4 recovered.

I have omitted 3 fatal cases; namely, one patient who had been ill three weeks before entering the Hospital and in whom perforation occurred the day after admission, another who was also the victim of advanced pulmonary tuberculosis, and a third who died of diphtheria when apparently safely through the attack of typhoid. On the other hand, I have included a patient who died of hemorrhage four days after admission and had been ill outside the Hospital for 21 days with insufficient attendance; the other fatal case of hemorrhage was also ill with fever a fortnight before he came into the Hospital, and, further, several of the patients had been ill for periods varying from 10—21 days before admission, during which time they had either been getting about, or had been inefficiently nursed, circumstances which materially add to the gravity of the prognosis.

Of these 70 cases, 5 died,—2, mentioned above, of hemorrhage, 2 of perforation, 1 during a severe relapse in the sixth week of a protracted attack, and the fifth of heart-failure: this last was a very bad case, the patient being admitted in a state of violent delirium with large subcutaneous hemorrhagic extravasations on the right buttock and both heels, and a petechial eruption on the skin.

The mortality is thus 7.1 per cent.: the duration of pyrexia in Hospital was 17 days; there was diarrhœa in 40 cases; hemorrhage in 6, which was fatal in 2; a true relapse occurred in 9 cases, the average duration was 13½ days; there was lobar pneumonia in 2 cases; thrombosis of the left femoral vein in 1, and of both femoral veins in another case.

It seems to me that until we have at our disposal a remedy which is capable of destroying the typhoid bacillus and neutralising its toxic products, no form of treatment is likely to reduce the death-rate below 6 or 8 per cent. in a fever which runs such a long course as enteric fever, exposes the patient to so many and such formidable complications, and exercises so great a drain on the patient's strength, and that the combined treatment by some form of intestinal antiseptic—innocuous to the patient in other ways—and by the employment of cold baths is at present the best available one. The low mortality now an established fact for the bath treatment is, in comparison with the results of other methods of treatment, a sufficient argument in its favour. Lastly, there is a striking improvement in the appearance of the patient treated by intestinal antiseptics and baths: the "typhoid aspect" is far more seldom seen, and never in so pronounced a degree as under the "expectant" plan; the pulse is better, with less tendency to dicrotism; the tongue cleans early and remains moist; there is less of the low, muttering delirium and of the trembling and twitching of the muscles; whilst the mind remains clearer, and sleep is more refreshing. In the absence of a specific remedy, all measures must fail in a certain proportion of cases; the object must be to make this unavoidable percentage of deaths as low as possible, and I believe that treatment directed on the lines above described is that which at present conduces most to this result.

---

In the discussion which followed Dr. Clarke's paper, Dr. SHINGLETON SMITH said a mortality of 7.1 per cent. was remarkably good for a long series of hospital cases. He had himself reported a mortality of 8.2 per cent. in a series of 195 cases. As regards antipyretic treatment, he agreed with Dr. Clarke that the cold bath was most valuable; yet the greater number of mild cases (119 in a series of 176 recoveries) required no antipyretic treatment: of the remainder only a small proportion required the use of the cold bath systematically, but phenacetin in conjunction with sponging would often prevent the necessity for more disagreeable measures. He admitted that we must sacrifice some degree of safety if we do away with the discomforts of systematic bathing. As regards intestinal antiseptics, he concurred in the utility of a few doses of calomel in the first week, but he was bound to adopt the view that "a general antisepsis, a germicidal influence upon bacteriological forms diffusely implanted in lymph tissues throughout the organism, is a vain fancy wholly unsupported by facts, and that the parasite is more resistant to such influences than the host." (Wilson.) He believed that the peptonising of the milk was a much more useful means of preventing and counteracting flatulent distension than any form of intestinal antiseptic, none of which appeared to prevent relapses, although they did something by preventing fermentation and over-distension of the bowel. As regards the treatment of the discharges from the body, he pointed out the necessity for prolonged exposure to the fluid if disinfection is to be adequate, and he thought that the reception of the fæcal discharge into cotton-wool or sawdust and the burning of the product should be carried out, and further, that, inasmuch as typhoid bacilli are frequently present in the urine, some disinfection of this fluid should be systematically adopted. As a result of the recent epidemic he had learned that digitalis must be used with caution, as it seemed to produce its physiological effects much more easily than in cases of pneumonia. He believed that the details of management were of such importance as to determine the life or death of the patient.—Dr. J. G. SWAYNE gave his own experience of enteric fever forty-five years ago, when he was attended by Dr. Symonds and Dr. W. Budd. Dr. Budd attributed it to well-water contaminated by a cesspool between York Place and Richmond Terrace, a place that in previous years had been a *habitat* of enteric fever. The fever set in with severe headache, which was soon relieved by twelve leeches to the temples; after this he took five grains of quinine *ter die*. After three weeks he (Dr. Swayne) began to improve, and left his bed at the end of a month.—Dr. E. MARKHAM SKERRITT believed the external application of cold was the best antipyretic in typhoid fever, yet in some cases phenacetin answered well; and quinine in single large doses of thirty or forty grains had the great advantage of securing a lower range of temperature for a considerable time—perhaps six or eight, or even twelve, hours. While the cold bath would give the best results in some cases, it was his usual practice to employ sponging or wet-packing as sufficiently effectual. The intestinal antiseptic which he used was hydronaphthol, conveniently given in tabloids, but only in those cases where the abdominal symptoms indicated definite local disturbance. The present epidemic had afforded many examples of absence of diarrhœa, pain, and tenderness and all abnormal abdominal distension; and the routine administration of antiseptics in such cases might lead to the error of attributing to treatment the favourable symptoms that were in reality due to the type of the attack. During the whole of the febrile stage the food should consist only of milk (which might be peptonised), and perhaps eggs beaten up, and beef-tea or some of the liquid preparations of meat. This diet should be adhered to for at least ten days after the temperature became permanently afebrile, and then soft food might be carefully added—no actual solids being allowed until a fortnight of apyrexia had elapsed. Want of care in dieting might induce relapse.—Dr. D. S. Davies claimed attention for that part of treatment which related, not to the safety of the patient, but to the safety of others, especially of those in immediate attendance upon the patient, and instanced the by no means infrequent attack of nurses or of students; the observance of an almost exaggerated cleanliness, especially in regard to the hands after attention to the patient, was insisted upon as absolutely necessary for safety, and the provision of ample means in wards or sick rooms for the immediate disinfection of hands or of soiled articles was pointed out to be essential. The choice of one germicide before another he considered of less importance than the care that whatever germicide was used should be in sufficient strength, and should be applied for so long a time as to be able to exert its full chemical destructive action upon each part of the dejection. Not the fæces alone, but the urine, the sputum, and in fact everything discharged from the patient must be dealt with as infective. The perchloride solution, containing half an ounce of perchloride of mercury dissolved in one ounce of crude hydrochloric acid, coloured with five grains of aniline blue, and mixed with three gallons of water (equal to 1 in 1000), might be kept in bulk and mixed as required with advantage, but the true principle of dealing with such infectious discharges was to mix with carbolated sawdust or other absorbent and burn at once, or, where the apparatus was available, to sterilise in a steam sterilising apparatus as designed and used at Newcastle-on-Tyne. In a sewered town, dejecta disinfected by chemicals as above may best be disposed of by throwing down properly constructed drains; but in country districts, if burying was resorted to this should be done with the utmost care, as recent experiments have shown that in unsterilised earth contaminated with refuse the bacilli die down in the winter only to multiply freely in the following summer. The specific infectivity of the stools may be long retained in convalescent patients, *e.g.*, in the milk epidemic of typhoid in Redland in 1879 the stools of a patient sent to a farmhouse during convalescence, discharged into a leaky privy, infected the pump, and thence the milk-supply, and gave rise to a serious outbreak.—Dr. C. Steele said that, thanks to Dr. William Budd, we know the pathological progress of the disease. He pointed out the necessity for quietude and liquid diet. Tepid sponging he had found to bring down the temperature, accompanied by a sense of great relief, and often sleep. To relieve fever, a few drops of tincture of aconite in a tumblerful of water are highly appreciated by patients, and induce gentle perspiration. Salol he had tried with some benefit; bicarbonate of soda and liquor pancreaticus (Benger) were of great use. For distension of the stomach diastol had proved effective.—Dr. H. Waldo said he admitted that the diet he gives in typhoid fever is more generous than formerly, but it is all fluid. Milk is the most suitable diet, and it leaves the least residue to form fæces. He allows the yolks of one or two eggs in twenty-four hours, and in the beef-tea or mutton or chicken broth fresh vegetables are boiled *and strained*. Coffee, Savory & Moore's peptonised cocoa and milk, and sometimes tea may be given, and he had never seen any harm arise. This dietary is more nutritious, and offers some variety. Five or six days after the temperature is normal, boiled tripe, or fish, or chicken, with bread, may be given. An initial dose of calomel, based on the view that the bacterial growth is chiefly in the intestine, is quite worthless, as the bacilli are not found in the stools until the end of the second week. They multiply in the intestinal walls, in the mesenteric glands, and in the spleen. If, as in cholera, the bacilli developed and produced the poison in the intestinal contents, there might be some reasonableness in this method. As an antiseptic he preferred chlorine gas dissolved in water, which not only reaches the intestinal lesions, but is absorbed into the blood. One of the best intestinal antiseptics is animal charcoal; it checks diarrhœa and lessens a distended abdomen. As an antipyretic, he strongly believed in baths. He had seen much harm done by antipyretic drugs, as phenacetin, as they depress the heart's action. For intestinal hemorrhage he advocated the use of turpentine in small and frequent doses.

The discussion was resumed on Jan. 12th, 1898.—Dr. C. ELLIOTT thought that the cold bath was the best mode of treatment; but there was one drawback to its use, namely, that it was impossible of general adoption in private practice. He thought the combined use of the wet pack and cold effusion gave equally good results, and was capable of universal application. This method not only reduced the fever, but calmed the nervous system and promoted sleep.—Dr. A. J. HARRISON said that one of the most important considerations was *rest* in bed. In 1868 he saw a large number of cases of a severe hemorrhagic type, and in these dilute nitro-muriatic acid and compound tincture of bark seemed efficacious. Of hypnotics, phenacetin proved very useful sometimes, and in a few desperate cases hypodermic injection of small doses of morphia, guarded by atropine, was probably efficacious in saving the life of the patient. Many mild cases require no drug treatment whatever.—Dr. B. M. H. ROGERS thought that the use of intestinal antiseptics was indicated to arrest if not to prevent the septic condition which was always more or less present in cases of enteric fever. We cannot arrest the growth of the bacillus, or prevent the absorption of the enteric toxin, but by antiseptics we might make the patient's condition more comfortable and prevent some of the complications which arose from the putrefaction of the intestinal contents. His practice was to give an initial dose of calomel and four-hourly doses of salol. Stimulants he reserved for the later stages, when the pulse showed signs of flagging or became intermittent. The use of cold baths was possibly good—Dr. Clarke's statistics showed it to be—but the difficulties in carrying out in private this method of treatment militated against its universal adoption.—Dr. WATSON WILLIAMS, who was unable to be present, sent some notes, in which he said: During the pyrexial period the food should consist mainly of milk, beef-tea, chicken or mutton broth, or beef-jelly. But even during this stage it is possible to introduce a grateful variety in the diet, if the patient be not too ill to appreciate such variation, by allowing a little coffee, milk-shape made with isinglass and flavoured with coffee or vanilla, junket, etc., and I have never seen any harm from allowing Mellin's or Benger's food. If the temperature is ranging low and the tongue is clean and normal, and there is no diarrhœa or tympanites, I have never observed any harm result from adding beaten eggs, cream, and, as soon as the temperature is normal, very finely minced or pounded chicken to the diet, while the patient's strength has been the better maintained in my experience, so that when a relapse occurs it is better borne. There is good reason to believe that so-called "solid food" in the form of pounded chicken is perfectly fluid long before it reaches the affected portion of the intestines, and that it is certainly as much a fluid as ordinary milk is after ingestion. We are all aware that milk may become converted into large solid masses in the stomach about as indigestible as anything that can be imagined. And when we find that milk is not well borne, we peptonise it or add lime water or barley water. But I have yet to hear it stated that we must give nothing but *peptonised* milk, because it is safer in *some* cases. Some cases so treated will relapse, but unfortunately they relapse no matter how strictly we keep on the so-called side of safety; and in my experience they relapse just as often, but with less strength to cope with a fresh attack and with greater liability to succumb to secondary complications. I do not remember ever having had a case in which relapse could in any way be traced by an unbiassed observer to such diet as I have allowed. Alcohol, and all forms of stimulants, I always withhold till the febrile stage is passed unless the pulse is weak and irregular and the heart flagging, or the patient shows indications of nervous exhaustion. Equally am I averse to the too free exhibition of digitalis or strychnine, and I may add that the more severe the case appears to be, the more anxious am I to defer prescribing, until absolutely necessary, any remedy which acts by using up potential energy, which the patient can ill spare. In dealing with pyrexia, I usually note whether the temperature ranges at 103° F. or 104°, or over, and give directions that the patient is to be sponged with water at about 80° F. when the temperature reaches 103° or 104°, as the case may be. Often enough warm sponging answers just as well by dilating the superficial arterioles and increasing the rate of loss of heat. Cold baths I have abandoned for several years, except for very high temperatures. Cold baths, ice-packing, and cold sponging remove heat by physical means, but they do not lessen the *production* of heat, in other words the disintegration of the tissues, they even tend to increase it. Yet, with the higher range of temperature, especially in some patients, the general condition calls for antipyretic treatment and is materially improved thereby, but cold baths are rarely advisable, I think, unless the temperature exceeds 105° F. I find that I have notified thirty-two cases within the last three months, Excluding five abortive cases and two which passed out of my care before they became severe, twenty-five cases of enteric fever of a more or less ordinary type have all recovered and gone away either well or practically well. But these cases have not been treated by any rule of thumb. Each has been treated on its merits, and certainly I have not adhered to the milk and beef-tea diet that is too much regarded as an axiom in dealing with enteric fever. The several advocates of the routine cold-bath or other antipyretic method; of the intestinal antiseptic method, or of the expectant or *safe* method, will each and all claim to have materially reduced bad complications and relapses and to have obtained the best results. There is indeed an element of truth in their contentions, but I do not feel convinced as to the decided advantage of the cold-bath method with which Dr. Michell Clarke has obtained such very good results, and which are worthy of warm congratulation. — Dr. G. PARKER asked if any value could be placed on Wright's preventive "vaccination," which had already undergone some testing. He thought the value of baths had been shown by the cases recorded of late years, and especially in the Brisbane records. Still there was a large mortality from perforation and hemorrhage which baths did not touch. Had we any intestinal antiseptics which could prevent this class of deaths? He thought the bath was not primarily valuable for the worst cases. The use of baths was chiefly to promote elimination of toxins, and to cause leucocytosis. If the latter occurred naturally under the influence of typhoid, it is possible that the human organism would be immune against typhoid, but it does not. Hyperpyrexia might be controlled by large doses of quinine, pyrexia itself reduced the multiplication of the bacillus some fifteen per cent., hence the seven lives in a hundred saved by baths were due rather to increased excretion of the poison than to lowered temperature. As to diet, while safety was clearly with liquid, and especially peptonised, food, it might be possible by giving grape sugar to prevent our patients from becoming mere skeletons, for Hale White showed that the great burning up of the proteids of the body might be prevented by its administration, and the proteids shielded by it.—Dr. F. H. Edgeworth remarked that whilst the cold-bath treatment in typhoid fever caused great diminution in the mortality, the systematic administration of internal antiseptics had very little influence on the death-rate. One important result of giving antiseptics in typhoid fever was that diarrhœa was checked if present; they often produced constipation. Now, *post-mortem* examination of several of the fatal cases in the recent epidemic showed that, contrary to received opinion, most extensive ulceration of the large intestine might be present. Such a condition might easily result in perforation under the mechanical distension of an enema. It was therefore better, in a case without diarrhœa, to omit antiseptics rather than to administer them and have to employ enemata. In controlling diarrhœa, antiseptics were infinitely preferable to narcotics or astringents. He advocated the use of junket, broken up by a spoon into small fragments, as a food in typhoid fever. Cow's milk, the ordinary diet in typhoid, was apt to coagulate in large masses in the stomach, and these not only mechanically irritated the intestine but were excellent seed-beds for the multiplication of micro-organisms until dissolved by the gastric and intestinal ferments. Such a food as broken-up junket was free from these unavoidable defects of raw or boiled milk, and gave excellent results both as regards gastro-intestinal flatulence and diarrhœa in typhoid fever.—Dr. J. O. Symes said that the routine cold-bath treatment had been abandoned in the Hospitals of the Metropolitan Asylums Board and in the London Fever Hospital.—Mr. J. Ewens believed it to be absolutely impossible to fully carry out the cold-bath treatment in general country practice, especially amongst the poor. He had found the sulpho-carbolate of sodium useful as an antiseptic in cases where diarrhœa was severe. Blisters (small and repeated) to the abdomen and internal administration of ol. terebinth were useful in tympanites. —In reply, Dr. Clarke said that speaking generally the discussion had confirmed the views which he had advocated in his paper. He thought the evidence in favour of the cold-bath treatment was very strong, and could not be lightly passed over. At the same time he fully admitted the difficulties which lay in the way of carrying it out and often rendered it impossible; not the least of these was the strong objection which the patients frequently had to the baths, and which in a nervous and excitable patient might render it extremely unwise to persevere with them. He agreed in the opinion expressed by many speakers against a routine treatment of the disease, and by no means wished to advocate routine treatment, but would modify it according to the indications in each case. At the same time he thought that those who too rigidly followed the "expectant method" were themselves in danger of falling into a routine. With regard to the objection raised by some speakers that antiseptic medication did not combat the specific organism of typhoid, he had been careful to point out that no such result was to be expected from the employment of the antiseptics advocated, but that their usefulness lay in controlling and preventing excessive putrefactive processes in the intestines.

---

